# New Development of Membrane Base Optoelectronic Devices

**DOI:** 10.3390/polym10010016

**Published:** 2017-12-23

**Authors:** Leon Hamui, María Elena Sánchez-Vergara, Rocío Sánchez-Ruiz, Diego Ruanova-Ferreiro, Ricardo Ballinas Indili, Cecilio Álvarez-Toledano

**Affiliations:** 1 Facultad de Ingeniería, Universidad Anáhuac México, Av. Universidad Anáhuac 46, Col. Lomas Anáhuac, Huixquilucan C.P. 52786, Estado de México, México; chiosanchezr@gmail.com (R.S.-R.); ruanova97@gmail.com (D.R.-F.); 2 Instituto de Química, Universidad Nacional Autónoma de México, Circuito exterior s/n, Ciudad Universitaria, Coyoacán, México D.F. 04510, México; ricardoballinas1989@gmail.com (R.B.I.); Cecilio@unam.mx (C.Á.-T.)

**Keywords:** nylon membrane, optical properties, electrical properties

## Abstract

It is known that one factor that affects the operation of optoelectronic devices is the effective protection of the semiconductor materials against environmental conditions. The permeation of atmospheric oxygen and water molecules into the device structure induces degradation of the electrodes and the semiconductor. As a result, in this communication we report the fabrication of semiconductor membranes consisting of Magnesium Phthalocyanine-allene (MgPc-allene) particles dispersed in Nylon 11 films. These membranes combine polymer properties with organic semiconductors properties and also provide a barrier effect for the atmospheric gas molecules. They were prepared by high vacuum evaporation and followed by thermal relaxation technique. For the characterization of the obtained membranes, Fourier-transform infrared spectroscopy (FT-IR), scanning electron microscopy (SEM), and energy dispersive spectroscopy (EDS) were used to determine the chemical and microstructural properties. UV-ViS, null ellipsometry, and visible photoluminescence (PL) at room temperature were used to characterize the optoelectronic properties. These results were compared with those obtained for the organic semiconductors: MgPc-allene thin films. Additionally, semiconductor membranes devices have been prepared, and a study of the device electronic transport properties was conducted by measuring electrical current density-voltage (*J-V)* characteristics by four point probes with different wavelengths. The resistance properties against different environmental molecules are enhanced, maintaining their semiconductor functionality that makes them candidates for optoelectronic applications.

## 1. Introduction

Optoelectronic devices, widely used in the semiconductor industry, are sensitive to environmental conditions, and therefore their operation is compromised. In order to mitigate this, an effective protection of the semiconductor materials against environmental conditions is necessary. The permeation of the water molecules into the device structure induces degradation of both the electrodes and the semiconductor itself. Metallophthalocyanines (MPcs), as organic semiconductors, have shown outstanding electronic and optical properties for optoelectronic materials, which is a consequence of their electronic delocalization [1,2,3,4,5,6]. These properties include, as well, photosensitivity, flexible structural modification and strong absorbance in the 600–800 nm region [4,7,8]. Also, it is known that the bandgap energy between HOMO (Highest Occupied Molecular) and LUMO (Lowest Unoccupied Molecular Orbital) can be tuned for these materials, as well as for photoluminescence (PL) and light absorption [5,6,9]. MPcs, formed by certain type of substituents or functional groups in their structure, present great chemical stability and are insoluble in most solvents [10], but not in water [10,11,12]. For this reason, it is important to protect the MPc without affecting the charge transport property in this type of semiconductor. The use of polymeric membranes is an option that may solve the requirements of the semiconductor isolation against the environmental water. Polymer Nylon 11, also known as Rilsan^©^, is a polyamide that combines qualities of flexibility, high resistance to aging, and excellent impact resistance (even at high temperatures) in high water or humid environments [13,14,15]. These materials present a high melting point and relatively low flexural modulus and hardness compared to different thermoplastic polymers that make them good candidates for polymeric membranes applications, which result from the amide linkages in the chain. Additionally, there are other polymers with similar mechanical properties like polypropylene, polymethylpentene, ethylene tetrafluoroethylene, and polytetrafluoro ethylene [13]. These polymers do not present some of the three requirements needed for a suitable protection of the MPcs for optoelectronic devices: (i) chemical resistance to organic solvents and environmental agents like humidity, (ii) ability to form uniform thin films, and (iii) effective integration between the polymeric matrix and the MPc semiconductor particles. Poly(octyl methacrylate-*co*-vinylimidazole [10], polystyrene, polyacrylonitrile [11], and poly(arylene ether sulfone) [16] have been used in the manufacture of membranes along with MPcs; however, these membrane applications were not intended for optoelectronic devices. Therefore, the Nylon 11 offers a viable option for the manufacture of semiconductor membranes as described in our previous studies [17]. The amide linkages in the chain provide these properties, among others, through the hydrogen bonds, allowing a solid interaction between the chains. Also, molecules that create hydrogen bonds can penetrate and interfere in the inter-chain hydrogen bonds weakening the hydrogen bond network [17]. Due to Nylon low permeability it is frequently used as a bagging material during shipping or storage. The mechanism by which a material in a gaseous state passes through a solid material of cross sectional area *A* and thickness *d* and is related to a pressure difference among it is called permeation [18,19,20]. Thomas Graham measured the permeability intensity of some gases. He was the first one who described a solution-permeation model for polymeric membranes, and his research on porous membranes led him to record Graham’s Law of Effusion [21]. It has been observed by Schowalter et al. that Nylon has lower permeability for many gases in comparison to similar materials [18]. An exponential dependence of the permeation of some polymers upon the square of the permeating gas atomic radius has been reported [19,20,22,23]. Electrospun fibres, as a support material, have been used by Mafukidze et al. and resulted in a lack of mechanical strength, which polyamides membranes employed in this work may possess [11]. Therefore, introducing MPcs as molecular materials whiting a polymer, such as Nylon 11, could promote outstanding and effective properties in membrane applications and cause them to remain intact in the fiber [11,24,25,26,27,28,29]. We report in this communication the fabrication of semiconductor membranes consisting of MgPc-allenes particles dispersed in Nylon 11 films. These membranes combine polymers properties with organic semiconductor properties and also provide a barrier effect for the permeating water molecules. We describe the fabrication and characterization of the obtained membranes by Fourier-transform infrared spectroscopy (FT-IR) spectroscopy, scanning electron microscopy (SEM) and energy dispersive spectroscopy (EDS), UV-ViS optical absorption measurements, Photoluminescence (PL), null Ellipsometry, and electrical Current-Voltage (*I-V)* characteristics using four point probes that were used to determine the chemical, microstructural, and optoelectronic properties. A further study of the semiconductor membranes should be done in order to determine their performance under harmful environmental conditions and the degradation relation of their optoelectronic properties such as bandgap and conductivity, even though the semiconductor membranes’ photo-degradation was analyzed by accelerated conditions during 8 h of light exposition.

## 2. Materials and Methods 

### 2.1. Membrane Preparation and Characterization

**Figure 1:**
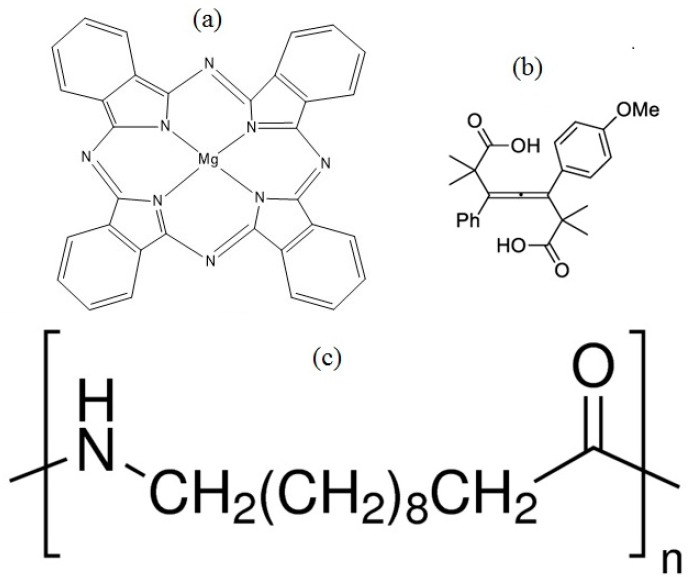
Structure of (**a**) MgPc, (**b**) tetrasubstituted allene, and (**c**) Nylon 11.

The Magnesium Phthalocyanine (MgPc) may be used as *p* type semiconductor or hole transport layer [30]; however, if this layer is constituted only by MgPc molecules, there will not be a high concentration of charge carriers that allow obtaining a significant current density. As a consequence of the latter, allene molecules are introduced in the material, in such a way that they contribute to the generation of charge carriers. MgPc (Sigma-Aldrich, Saint Louis, MO, USA) and Nylon 11 (Sigma-Aldrich, Saint Louis, MO, USA) (Figure 1a,c) were obtained from commercial sources, without purification prior to their use. The powders for the allene C_24_H_26_O_5_ (Figure 1b) were synthesized using the procedure reported previously by some authors [31,32]. The semiconductor membranes were manufactured from the following steps: (i) doping and characterization of MgPc; (ii) deposit of consecutive layers of Nylon 11 polymer matrix and doped MgPc; (iii) thermal relaxation treatment, for the incorporation of semiconductor particles in the membrane matrix; and (iv) structural characterization.

These stages are described as follows: (i) The MgPc doping took place by reflux in absolute methanol for three days, using a 1:0.1 MgPc-allene ratio. 39.4 mg (0.1 mmol) of C_24_H_26_O_5_ in 10 mL of absolute methanol were added to a 30 mL solution with 536 mg (1 mmol) of MgPc. The final solution was kept in reflux, and later it was filtered to obtain the doped semiconductor, washed, and vacuum dried. The product was characterized by Infrared spectroscopy (IR) (Thermo Fisher Scientific Inc., Waltham, MA, USA) and also for its melting temperature; the characteristic FT-IR signals corresponding to the main functional groups are reported in Table 1. (ii) Semiconductor membranes consisting of doped MgPc particles embedded in Nylon 11 matrix were prepared by the consecutive sublimation at 573 K of the polymer and the doped MgPc in a high vacuum chamber. The vacuum in the chamber was achieved by the operation of two pumps: a mechanical pump that generated an initial vacuum of 10^−3^ Torr, and a turbo-molecular pump that allowed a deposition pressure in the chamber of 1 × 10^−6^ Torr. The deposit rate was the same in all cases (1.4 Å/s). The membranes were deposited over different types of substrates: high resistivity monocrystalline n-type silicon wafers (c-Si), quartz, Corning glass, and indium tin oxide (ITO)-coated glass slides previously washed with different solvents in ultrasonic bath and vacuum dried. The same deposition system was used to obtain all samples, with the substrates arranged in similar geometries and a tantalum crucible. Throughout the deposition processes, the thicknesses were monitored using a quartz crystal monitor and, after the deposit, IR spectroscopy in the membrane was performed to verify the main functional groups. (iii) In order to embed the MgPc particles into the Nylon 11 matrix, the membranes were treated by thermal relaxation in air, at 413 K, for 10 min, and were analyzed again by IR spectroscopy. These was done to verify the incorporation of semiconductor particles into the matrix and also to determine the crystalline form present in doped MgPc. The MgPc shows two main crystalline forms: alpha (α) and beta (β), which can be verified at 777 and 720 cm^−1^, respectively [33,34,35,36,37]. Furthermore, to evaluate the effect of Nylon 11 on the semiconductor, doped MgPc and intrinsic thin films were deposited additionally. The FT-IR analysis was performed for the intrinsic MgPc, doped MgPc, and membranes on a Nicolet spectrometer (Thermo Fisher Scientific Inc., Waltham, MA, USA). (iv) To carry out the characterization of the membranes, a Bruker microanalysis system was coupled to a ZEISS EVO LS 10 scanning electron microscope (SEM) (Cambridge, UK). This equipment was operated using quartz substrate with a 20 kV voltage and a 25 mm focal distance.

### 2.2. Electrical and Optical Measurement

A glass/ITO/Nylon11: MgPc-allene/Ag type sandwich structure was prepared in order to evaluate the semiconductor behavior of the membrane. For the electrical characterization of the membranes, a programmable voltage source, a sensing station with lighting and temperature controlled circuit from Next Robotix (Comercializadora K Mox, S.A. de C.V., Benito Juárez, Mexico, Mexico), and an auto-ranging Keithley (Tektronix Inc., Beaverton, OR, USA) 4200-SCS-PK1 pico-ammeter were employed. The optical absorption was measured on a Unicam spectrophotometer (Thermo Fisher Scientific Inc., Waltham, MA, USA) model UV300, in the wavelength range of 1100–200 nm. The Tauc optical gap was determined from the absorbance in membranes at different wavelengths of the UV-Vis spectrum and compared to that obtained in doped and intrinsic MgPc, in order to review the effect of the Nylon 11 in the semiconductor behavior of the material. Additionally, a Gaertner L117 Ellipsometer (Gaertner Scientific Corporation, Skokie, IL, USA) equipped with a He-Ne laser (λ = 632.8 nm) was used to measure the refractive index and to verify the thickness obtained from the evaporator quartz microbalance. Photoluminescence (PL) was measured using a He-Cd laser (Kimmon Koha Co., Ltd., Centennial, CO, USA) with an excitation wavelength of 325 nm and integration time of 100 ms. Finally, in order to evaluate the degradation effect of the radiation on the membranes, an irradiation of a 360 watts, 82 V Bias Dukane 28A653A lamp was used for 8 h. Following the accelerated radiation test, the *I-V* behavior in membranes was evaluated again. It is important to mention that the analysis over the electric behavior was performed in terms of Current Density-Voltge (*J-V*), using a membrane cross section area of 3.75 cm^2^.

## 3. Results and Discussion

Due to drastic temperature changes during the deposition and the thermal relaxation that implies the manufacture of the membranes, the IR spectroscopy was carried out for intrinsic, doped MgPc thin films, and finally, for the Nylon 11 membrane (Table 1). With the aim of monitoring any chemical change in the material and beside the modifications in the crystalline structure of semiconductor, IR spectroscopy was made. The spectra for the MgPc include a band around 1610 cm^−1^ corresponding to C–C stretching vibrations within the macrocyclic ring. The band around 1333 cm^−1^ is assigned to the C=N vibrations in the macrocyclic ring, with around 1285 and 756 cm^−1^ assigned to the isoindole plane and C–N stretching vibrations, respectively, and around 1421, 1163, and 1117 cm^−1^ resulting from the interaction of carbon with the peripheral ring hydrogen atoms. On the other hand, the value around 3080 cm^−1^ corresponds to the vibration of the O–H bond of the carboxylic acid C(O)O–H in the allene, and around 2926 cm^−1^ likewise corresponds to the vibration related to the C–H bonds of the methoxy substituents. Finally, the bands around 3308 and 3077 cm^−1^ correspond to the stretching vibrations for N–H, and the band around 1652 cm^−1^ corresponds to the C=O vibrations of the polymeric matrix. The crystalline structure of the MgPc is another variant that can be monitored by IR spectroscopy, due to its different polymorphs forms [33,34,35,36,37]. The MgPc α-form and the β-form can be identified by a band around 720 cm^−1^ and a band around 778 cm^−1^, respectively [33,34,35,36,37]. The MgPc keeps its two crystalline structures α and β during the fabrication process of the membrane (see Table 1). Apparently by surrounding the semiconductor particles, the Nylon 11 inhibits the phase change, and, according to what is observed in Table 1, the doped MgPc did not suffer any chemical changes during the fabrication of the membrane. It is important to consider that, according to Jonathan Albo et al., polyamide membranes may be affected by the presence of gaseous molecules (water, gases, or solvents) [38,39]. The gas molecules’ size, polarity, and type of interactions generate these effects. The latter is the reason why, according to the application of the membrane, the polymer may need a pretreatment [38,39]. This pretreatment may include the cleaning of the membrane by solvents and the drying of the residual solvent that could be inside the structure of the polymer. A drying pretreatment at different temperatures can also be carried out followed by a drying process with solvents [38]. Considering the previous factors, in the present study the membrane was prepared by the physical method of Nylon11 sublimation at high vacuum. This process implies the heating of the polymer at 573 K, which generates the separation from the polymer of the water molecules and subsequently the elimination by the vacuum of the chamber. Additionally, the thermal process for the incorporation of MPc into Nylon 11 is carried out at 413 K; this temperature favors the elimination of water molecules that can remain within the membrane, and it is confirmed by the IR spectrum [40]. Furthermore, the sublimation at high vacuum and thermal relaxation of Nylon 11 was selected, unlike other membrane manufacturing processes, in order to avoid the presence of solvents that can interact with the polymer [10,11,12]. Therefore, we may conclude that the manufacturing process of the membrane from doped MgPc-Nylon 11 by high vacuum evaporation, and later by thermal relaxation, is appropriate.

**Figure 2:**
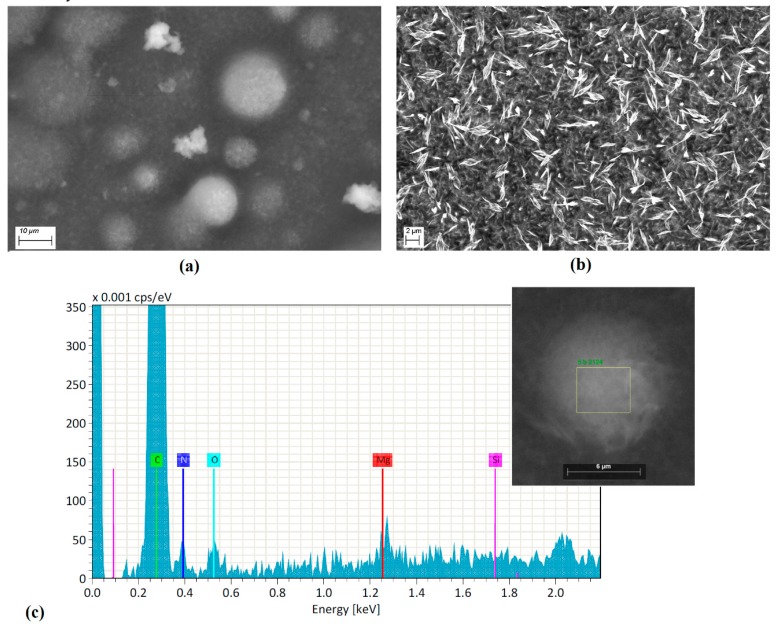
SEM image of (**a**) the particles within the membrane, (**b**) membrane matrix, and (**c**) EDS spectrum for particle in the membrane.

With respect to SEM Micrographs shown in Figure 2a, the membrane is observed to be formed by particles of agglomerated (most of them), with rounded structure of different sizes. During the deposition of the membrane on the substrate, the heterogeneous nucleation process generated by MgPc particles depends directly on the structure of the doped MgPc and the thermic gradient between the substrate (298 K) and the system doped MgPc-Nylon 11 (573 K). Apparently, due to the lower free energy per unit volume of the sphere, the nuclei of the membrane deposited on the substrate resemble this geometry that, when it grows, is maintained as the deposit advances and the membrane is formed. Inside the membrane are also present particles of amorphous doped semiconductor (Figure 2a) surrounded by Nylon 11, which generates a heterogeneous morphology. It is worth mentioning that the polymeric matrix is evenly distributed over the substrate (Figure 2b); this uniformity prevents the dissipation of the electromagnetic radiation that may affect the electrical behavior in the doped MgPc. Thermal relaxation is also an aspect to consider; while heating Nylon 11, fibers undergo an elongation, which allows MgPc doped particles to get into the matrix of the membrane. As the polymer gets cold, it is followed by a contraction of the fibers, and the semiconductor particles remain surrounded by contacted fibers (Figure 2c). In addition, EDS (Bruker Corporation, Harvard, MA, USA) studies were carried out on the deposited membranes over Corning glass. In Figure 2c, the presence of different atoms in the semiconductor particles and covered by the Nylon 11 matrix that integrate the membrane is observed. The presence of the magnesium is correspondent to the MPc molecule; nitrogen can be an indication of both the presence of the molecule itself, as well as of Nylon 11, and oxygen is present in both the polymer and the allene. By complementing this information with that reported by IR spectroscopy, it is confirmed that nitrogen is present in the macrocycle by the presence of the bands assigned to the C=N and to the C–N vibration modes. This is also observed in Nylon 11, by the presence of the band referent to the stretching vibrations for N–H. The presence of oxygen in the Nylon 11 is verified by the signal referent to C=O vibration, and in the allene, its presence is confirmed by the band O–H observed in the carboxylic acid.

**Figure 3:**
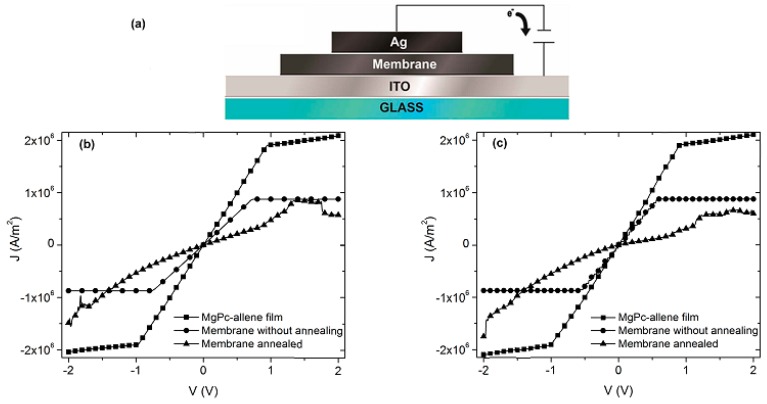
(**a**) Schematic structure of the electrical measurements in the membrane and thin film. *J-V* characteristics in (**b**) natural light conditions and (**c**) darkness conditions where ITO is positively biased.

The MgPc is an organic semiconductor π-conjugated that falls within the category of a discotic system formed by an aromatic nucleus. It has the capacity to organize itself in almost mono-dimensional columns, which allows the charge transport in one direction only. This one-directional charge transport is influenced by the π-stacking interactions between neighbor molecules, and it can be evaluated by the four point probe collinear method. Since the charge carrier density inside the membrane is too low for the transport in the interior, it is necessary to inject charges from an electrode that acts as anode. In this case, a transparent conductor contact of ITO was used to inject holes, while a silver electrode as cathode was used to inject electrons, both as a consequence of the application of an electric field (see Figure 3a). *J-V* characteristic was evaluated in natural light conditions (Figure 3b), as well as in darkness (Figure 3c), in order to verify the electric behavior of the membrane before and after the thermal treatment. Both light conditions were used to verify the electromagnetic radiation effect generated on the membrane, which in turn is necessary to establish the type of optoelectronic device that can be used. In Figure 3b,c, it is observed that the behavior is practically similar: the radiation has no effect in both the semiconductor thin film and the membrane. On the other hand, important differences in the electric behavior of the membrane before and after thermal treatment were observed. Before thermal treatment, the membrane presents a symmetric behavior, similar to the one of the semiconductor thin film. Additionally, at low voltages the behavior is ohmic, while at voltages higher than 1 V a change of slope is shown, referring to an accumulation of charges that are similar to a resistor. After the thermal treatment, with the incorporation of semiconductor particles in the Nylon 11 matrix, a behavior more likely to a light emitting diode is observed [30]. Apparently, the hole injection is conducted through the ITO, and the electron injection through the Ag. These carriers travel from the electrodes to the membrane, where, apparently, light is produced by the radiative recombination of generated excitons from the injection of the carriers [30]. It is evident that the thermal relaxation of the membrane generates a change in the electric behavior; however, it is necessary to study this membrane under different electromagnetic radiation conditions.

**Figure 4:**
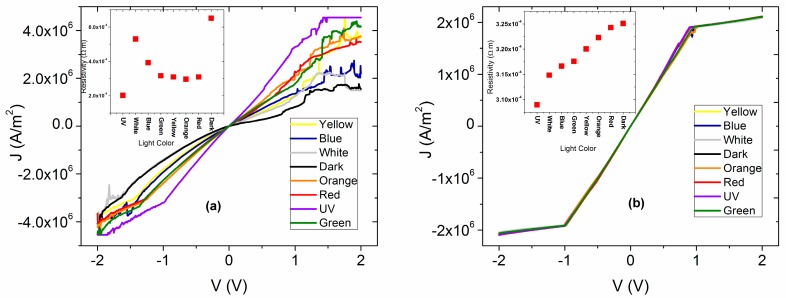
*J-V* characteristics of (**a**) annealed membrane and (**b**) semiconductor film where ITO is positively biased.

The electrical characterization of the membrane that was thermally treated was carried out by measuring the *J-V* curves in artificial and natural lighting conditions. The latter simulates the irradiation of the membrane with sunlight and in dark conditions with the aim of evaluating the membrane functionality that is analogous to a light emitting diode. The results are shown in Figure 4a, where it appears that under the presence of different wavelengths of electromagnetic radiation, the membrane shows practically the same behavior. Although, the high energy UV radiation generates the higher density of current transported to the interior of the membrane, while in darkness, the current density drops considerably. Additionally, with respect to the *J-V* curves measured under illumination conditions, ohmic behavior is observed, whereas the fact that such low current density in dark conditions was obtained is indicative of high resistance at the interior of the membrane. This resistance can be associated with the presence of Nylon 11, and, as a consequence, the electric behavior of an annealed thin film of doped MPc without Nylon also was analyzed (Figure 4b) in order to be compared. For voltages lower than 1 V, an ohmic behavior is observed, but when the voltage increases, a change of slope is observed due to an alteration in the regime of charge transport mainly associated with the accumulation of charges inside the semiconductor film. This behavior is present in both measurements performed in dark, as well as under illumination conditions. It is concluded that the presence of Nylon 11 enhances the electric characteristics in the material, although at voltages below 1 V, the behavior is practically ohmic in both the semiconductor without the Nylon and as membrane. By increasing the voltage, a charge accumulation is generated in the semiconductor film and can be controlled by a SCLC type current (space-charge-limited conduction) or also by a model based on cargo traps [41], while in the other hand, the membrane ohmic behavior is maintained under illumination conditions. Moreover, the presence of the Nylon 11 allowed a broad variation of the current density with the different incident wavelengths that is not observed for the semiconductor film itself.

On the other hand, in Figure 4a,b the electrical resistivity (*ρ*) under the effect of different types of illumination for both membrane and semiconductor film can be observed. It is important to notice that the calculated *ρ* is of the same order of magnitude in both cases and that under natural lighting conditions (white labeled in both Figures), *ρ,* between the film and the membrane, did not show significant variation. Therefore, apparently Nylon 11 does not generate an electric isolating effect in the membrane. In the range of visible radiation, there are changes due to the effect of polymer on the membrane. In contrast to the film, the *ρ* increases when the wavelength is increased due to the shift to low energy (blue radiation to red). On the membrane, the *ρ* increases as the wavelength of incident light is decreased from red to blue. However, both in the membrane and in the semiconductor film, the lowest *ρ* occurs under the presence of UV illumination. This is related to the density of transported charge carriers, which is superior for the highest energy of UV radiation where a higher generation of charge carriers is presented. The highest *ρ* is in dark conditions for both the membrane and the semiconductor film, where, apparently, the rate of generation of charge carriers is lower. It is important to mention that the electric resistivity is the inverse of the conductivity:

(1) σ=1/ρ

Based on the *ρ* values obtained for the membrane at room temperature and under ilumination conditions (Figure 4a), a σ in the range of 19.12 and 49.51 S/cm was obtained. The σ value of the membrane is in the range of the semiconductor materials (10^3^ to 10^−8^ S/cm) [30]. Thie results, as expected, are important for the application of the proposed membrane in the manufacture of optoelectronic devices. The latter is because it allows the electric conductivity value of the semiconductor to be maintained as integrated in the membrane. The characterization of the membrane in dark conditions reproduces behavior analogous to that of a simple light emitting diode (OLED). As mentioned above, the injection of charge carriers of opposite electric charges will flow from the electrodes to the membrane. Hereby, the recombination of a generated exciton, electrically from the injection of carriers, may produce light. The electric conductivity obtained for the membrane in dark conditions at room temperature is 15.27 S/cm; this value is also above the organic semiconductors range reported. It is known that the water molecules may reduce the electrical conductivity of the MPcs semiconductor due to its capability to dissolve the material [42,43]. The latter is amplified when high energy light is used for charge carrier generation and a diminution of the generated carries is presented. This is as a consequence of an increase of the trapping probability and an electron mobility decrease within the membrane [44]. As observed in Figure 4b, there is no such effect in the *J-V* characteristics, which in turn indicates that the semiconductor material is isolated from water molecules in the environment.

Additionally, the refractive index (*n*) was determined in order to get information about the change in direction/velocity of the electromagnetic radiation experiments, for different types of illumination (natural and artificial), when passing through the membrane. The results obtained by ellipsometry, for both membrane and semiconductor film, are shown in Table 2. It is observed that the refractive index of the membrane is lower than that of the film and is mainly related to the Nylon 11 fibers that surround the MgPc doped particles. The refractive index value for conventional Nylon 11 is 1.52, and the values obtained are lower than that [45]. Also, it can be observed that the values of both the film and the membrane are very close to the air refractive index (~1), which indicates that the transmition of the light across the mediums has a small deviation from its original direction and reduces the reflection of the interface between mediums.

**Figure 5:**
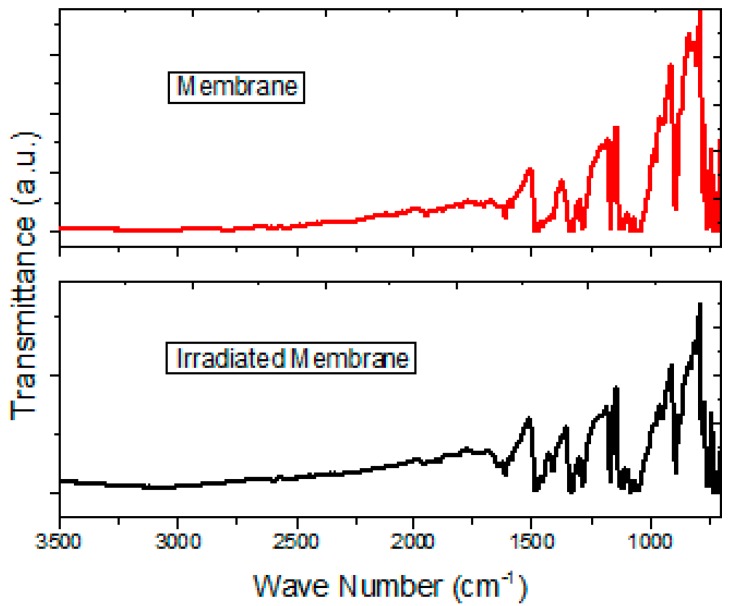
IR spectrum for membrane after and before irradiation.

In order to analyze the possible photo-degradation of Nylon 11 and its effect on the transport properties in doped MgPc, the membrane was irradiated with a 360 Watts lamp in accelerated conditions during 8 h; then, the presence of main functional groups was evaluated by IR spectroscopy, and finally the *J-V* behavior in darkness and under illumination was determined. This behavior was compared to that of the semiconductor film irradiated under the same conditions. In Figure 5 the IR spectrum of before and after the light irradiation is shown. The curves show signs referent to the stretching vibrations for N–H (around 3308 and 3077 cm^−1^) and the band assigned to the C=O vibrations (around 1652 cm^−1^) in the Nylon 11. Based on IR spectroscopy, it is possible to conclude that there was not a chemical decomposition of the Nylon in the membrane, nor of the doped semiconductor (the bands corresponding to C–C, C=N and C–N vibrations in the MgPc; the vibrations of the O–H and the C–H bonds of the allene are observed). It is worth mentioning that during the irradiation, the membrane was exposed to environmental conditions such as the presence of air and humidity of the environment. However, the presence of the band assigned to the O–H bond of water in the IR spectrum is not observed. Apparently, Nylon 11 functions as a suitable barrier between the semiconductor and the environmental water. It is worth mentioning the above, since most of the organic semiconductors tend to oxidation and degrade chemically in environmental conditions [11]. These cause them to lose competitiveness against the inorganic semiconductors like silicon; the fact that the Nylon protects the semiconductor favors the use of these membranes for optoelectronic devices, increasing, as well, its useful life.

**Figure 6:**
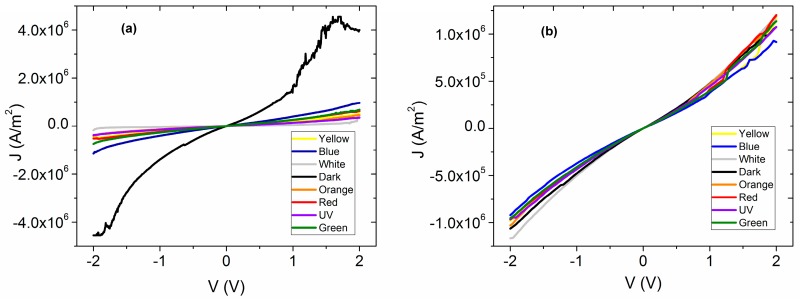
*J*-*V* characteristics of (**a**) membrane and (**b**) semiconductor film irradiated 8 h.

Respectively, the *J-V* evaluation was done in order to verify the effect of the irradiation on the electric properties of the semiconductor. It is observed in Figure 6 that, with the exception of the dark condition for membrane, both the film and the membrane itself show a similar behavior for the evaluated voltage range. The latter exists with no significant variation in the current density when the measurement voltage polarity changes. Although, a significant decrease of the current values in the membrane is observed with respect to that obtained before the 8 h of accelerated irradiation. Current density transported in the material is lower for the membrane, except in the case of dark condition, where, practically, the behavior does not change with respect to the one obtained before the irradiation. Besides, there is an increase in the current density that is transported when the voltage increases. Apparently, the membrane maintains an operation similar to that of an OLED [46,47,48,49]: the MgPc is used as *p* type semiconductor, and allene molecules are introduced in the material, in such a way that they contribute to the generation of charge carriers. Then, the presence of both compounds favors the injection and electron-hole transport within the membrane, facilitating the charge balance and their transport between the electrodes. It is worth mentioning that in the case of the semiconductor film the accumulation of charges in the material is reduced with the accelerated irradiation, although the *J* values do not exceed those obtained in the membrane. Based on above, it is observed that the presence of Nylon 11 in the membrane does not affect the electrical functionality of the semiconductor in its application to the manufacture of the OLED, and, according to IR spectroscopy, the polymer remarkably protects its functionality against degradation by the presence of humidity in the environment [11].

**Figure 7:**
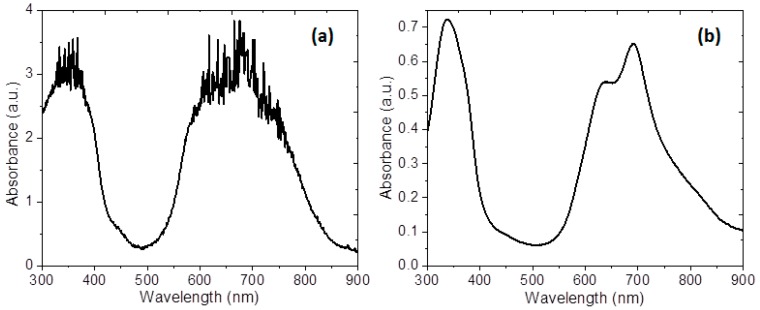
Absorption spectra in the range of 900–300 nm for (**a**) membrane and (**b**) semiconductor film.

The optical absorbance spectra of the membrane and the semiconductor film deposited on quartz were recorded for a wavelength range from 1100 to 200 nm and are shown in Figure 7. To characterize the optoelectronic properties of the membrane, optical absorption measurements were conducted to determine the parameters that describe the electronic transitions. The differences observed on the absorbance can be attributed to the presence of Nylon 11 in the membrane; the shape of the spectrum is due to MgPc with D_4h_ symmetry [50]. The spectral properties of the membrane are caused by the MgPc doped semiconductor, which presents two typical bands: the Q-band and the B-band; these bands confirmed the presence of MgPc doped on the surface of the membrane [11]. Two peaks for Q-band can be observed in the visible region: a high energy peak, around 640 nm, and a second one, a low energy peak, around 690 nm [6,32,33,34]. The high-energy peak of the *Q*-band is assigned to the first π–π* transition on the MgPc doped semiconductor. A second π–π* transition associated with the low-energy peak of the *Q*-band is explained as an excitation peak, as a surface state, and as a vibrational internal interval [33,34]. The appearance of a red shifted absorption at 640 nm relative to the monomer peak at 690 nm is explained as a result of coplanar association of MgPc macrocycles progressing from monomers and leading to aggregates [11,50]. The MgPc rings are arranged in a face-to-face position (H-type) in the aggregate [50]; however, according to Lapok et al. [50] in their work related to the use of MgPc on membranes, the spectra do not present information related to the α y β forms of the MgPc. The absorption spectrum of the monoclinic structure (α-form) has a doublet around 708 and 653 nm, while in the tricyclic spectrum of the (β-form) a high intensity band is observed at 646 nm with two shoulders at 620 and 665 nm [51]. Finally, the B-band is within the UV region of the spectrum, at around 340 nm [6,28,29,30], and it refers to the electronic *n-*π* transitions between the molecules. The B-band is due to $a_{2u}\left(\pi \right)\to e_{g}\left(\pi^{*}\right)$ together with $b_{2u}\left(\pi \right)\to e_{g}\left(\pi^{*}\right)$ transitions [52,53].

**Figure 8:**
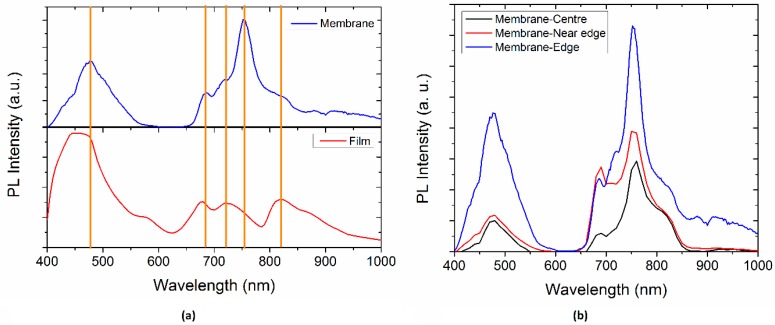
Photoluminescence spectra of (**a**) membrane and semiconductor film and (**b**) membrane at different positions.

Figure 8a shows the Photoluminescence spectra of the membrane and semiconductor film. The PL spectra were normalized to the thickness of each samples. Four peaks that the film and the membrane have in common can be observed: ~475, ~680, ~720, and ~820 nm. The short wavelength peak in both materials corresponds to the region of no absorption in Figure 6; however, small band tails are observed. The membrane shows another peak at ~751 nm that is characteristic of the Nylon 11 and disappears for the film. It is important to notice that for the semiconductor film (Figure 8b), the absorbance values are small compared to those of the membrane. The peak ~680 nm, associated with the π*–π relaxation transitions of the doped MPc macrocycle, is red shifted for the membrane. The last is related to the π–π* stacking of the conjugated MPc, as the semiconductor particles are introduced in the Nylon 11, which infers a molecule aggregation. The membrane narrow peak ~475 nm is mainly related to the Nylon 11 [54,55], which in turn is a doublet of the peak ~751 nm optically-allowed exciton recombination. Moreover, doped MPc also contributes to the PL intensity of the previous signal and is dependent on the electron transition to deeper levels [6,32]. The broadening of this peak for the semiconductor film may be associated with the Pc orbital overlap with the Mg central atom and with the amorphous nature that allows the formation of delocalized states between HOMO and LUMO. The latter, as a consequence, generates a non-radiative relaxation and it is responsible for the semiconducting properties. On the other hand, the semiconductor film shows a small peak ~590 nm attributed to the Pc [6] and PL emission at ~430 nm that is identified as singlet exciton recombination. Also, the PL in the visible range was observed with naked eye and varies in colour, red shifted for the membrane, as a consequence of the different peaks intensity. Therefore, a significant change is observed in the PL efficiency and PL maximum position when the MgPc are introduced in the Nylon 11 fibers.

The PL Spectra of the membrane at different positions within the sample are shown on Figure 8b. First, it can be appreciated that, as the measure is made closer to the edge of the sample, the PL spectra intensity is increased. The peak at approximately 720 nm is more pronounced at the edge. On the other hand, the peak at around 680 nm is sharpened, as the measure is made closer to the edge and it is more intense near the edge (red line curve). All of this indicates that the emission intensity varies along the membrane, but the emission wavelengths remain the same. This could be related to the amount of MgPc doped particles that are incorporated inside the fibers of Nylon 11 during evaporation. Substrate temperature during deposition might have a thermic gradient between the centre and the edge, which explains the increase in the semiconductor particles at the edge. Furthermore, the higher PL intensity could be attributed to the formation of charge transfer complex and to the arrangement of the molecules. It is worth mentioning that positions close to the membrane centre present a PL emission, with minimal variations indicating a homogenous material. 

The reflectance (*R*) percentage obtained from Equation (2) and the refractive index are shown in Table 2. The optical properties of both the semiconductor film and the membrane can be analyzed according to the Tauc model, as their estimated reflectance is lower than 15% [32,56]. This model is used to determine the optical properties of amorphous semiconductor materials (the presence of Nylon 11 in the matrix of the membrane gives an amorphous array to the structure). The low reflectance percentage allows a large number of photons with different wavelengths to be absorbed.

(2) $$R=\frac{100{\left(n-1\right)}^{2}}{{\left(n+1\right)}^{2}}$$

According to the semi-empirical Tauc model, the optical band gap (*E*_g_) can be deduced from the UV-Vis absorption spectrum [32,56]. In amorphous semiconductors, electronic transitions are described by non-direct transitions with no conservation of electronic momentum. The Tauc optical gap for non-direct transitions could be determined by the extrapolation to zero of the linear regions of the (α*hν*)^½^ = *f(hν)* plots. The absorption coefficient (α) is related to each wavelength that is irradiated in terms of the transmittance (*T*) and thickness (τ) of the sample. Film and membrane thickness were obtained by null ellipsometry measurements, as shown in Table 2.

(3) $$\alpha =-\frac{1}{\Gamma }\ln\left(T\right)$$

On the other hand, Equation (4) was used to calculate the photon energy (*E*_photon_) for each wavelength (λ), in which *c* is the speed of light in the vacuum and *h* is Planck’s constant. The optical gap band results are shown in Table 3, where it can be observed that the membrane presents practically the same optical band gap as the MgPc semiconductor doped with allene.

(4) $$E_{photon}=\frac{hc}{\lambda }$$

As expected, the Tauc’s optical gap is lower for the doped MgPc than for the intrinsic, and the optical gap is also higher for the membrane without the thermal treatment than for the membrane after the thermal relaxation. However, the little variation between the optical gap of the membrane and the semiconductor film is a sign of the viability for using the Nylon 11 membrane in optoelectronic applications, even though the final structure array of the membrane is amorphous. The structural disorder due to the weak non-covalent interactions that govern it leads to a not identical environment of each semiconductor molecule. The latter is compared to the other molecules integrated in the membrane, i.e., the energy of the molecules orbitals that form the membrane and cause the molecule to not be isoenergetic, but will present an energy distribution. However, the value obtained from Tauc’s optical gap for the membrane lets us conclude that the semiconductor properties of the doped MgPc were not lost. Comparing the Tauc optical gap shown on Table 3 to the PL maximum peak energy it can be found that, for the membrane, the indirect bandgap transition is related to the peak of ~475 nm. On the other hand, for the semiconductor film, an indirect bandgap transition is related to the peak ~427 nm.

## 4. Conclusions

Semiconductor membranes consisting of MgPc-allene particles dispersed in Nylon 11 were manufactured using a high vacuum evaporation technique followed by thermal relaxation. These two processes mainly avoid the presence of water molecules that may dissolve the semiconductor, due to the temperatures superior to 373 K. These semiconductor membranes combine polymer properties with organic semiconductor properties and, according to IR spectroscopy, also provide a barrier effect for molecules of environmental agents like water. When evaluating the electrical behavior of the membrane, before and after the thermal treatment, a significant difference in behavior is observed: while the membrane without treatment is similar to a resistor, the membrane that is thermally relaxed behaves like a diode. The ohmic behavior of doped MgPc in the membrane is still maintained with the presence of Nylon 11, even after being subjected to accelerated photo-degradation conditions. Moreover, morphological and structural changes should be studied to identify the cause of a probable performance difference in harmful environmental conditions. As the aim of this work is the development of membrane base optoelectronic devices, the latter is taken into account as a part of future work. According to IR and UV-Vis spectroscopy, the MgPc doped presents α y β crystalline form, with the MgPc rings arranged in a face-to-face position in the amorphous membrane. Its optical band gap was evaluated by Tauc’s model for non-direct transitions. The optical band gap slightly decreases with the introduction of the polymer, which indicates that the semiconductor characteristics of the MgPc-allene are maintained, although they are part of the membrane.

## Figures and Tables

**Table 1:** Characteristic FT-IR signals for MgPc intrinsic, doped, and membrane.

Sample	(C–C) [cm^−1^]	(C=N) [cm^−1^]	(C–H) [cm^−1^]	(C–N) [cm^−1^]	α-Form [cm^−1^]	β-Form [cm^−1^]	Nylon 11 [cm^−1^]	Allene [cm^−1^]
MgPc intrinsic (KBr pellet)	1609	1330	1420, 1163, 1115	1283, 752	723	775	-	-
MgPc-allene doped (KBr pellet)	1612	1335	1421, 1163, 1117	1285, 755	723	773	-	
MgPc-allene doped (thin film)	1615	1333	1420, 1163, 1115	1285, 754	724	776	-	3083, 2026
MgPc-allene/Nylon 11 membrane without thermal relaxation	1615	1332	1418, 1161, 1116	1282, 753	727	776	3308, 3077 1652	3083, 2929
MgPc-allene/Nylon 11 membrane with thermal relaxation	1616	1333	1420, 1163, 1114	1282, 753	723	777	3311, 3074 1654	3080, 2923

**Table 2:** Characteristic parameters of membrane and semiconductor films.

Sample	Thickness [nm]	Refractive index (*n*)	% Reflectance (*R*)
Semiconductor film	276.18	1.21	0.8659
Membrane	226.50	1.16	0.5426

**Table 3:** Non-direct Tauc optical gap.

Sample	Non-direct optical gap (eV)
MgPc	2.8
MgPc-allene film	2.1
Membrane without annealing	2.6
Membrane annealed	2.4

## References

[1] Casstevens M., Samok M., Pfleger J., Prasad P.N. (1990). Dynamics of third−order nonlinear optical processes in Langmuir—Blodgett and evaporated films of phthalocyanines. J. Chem. Phys..

[2] De la Torre G., Vázquez P., Agulló-López F., Torres T. (1998). Phthalocyanines and related compounds: Organic targets for nonlinear optical applications. J. Mater. Chem..

[3] Claessens C.G., Hahn U., Torres T. (2008). Phthalocyanines: From outstanding electronic properties to emerging applications. Chem. Rec..

[4] De Saja J.A., Rodríguez-Méndez M.L. (2005). Sensors based on double-decker rare earth phthalocyanines. Adv. Colloid Interface Sci..

[5] Hajri A., Touaiti S., Jamoussi B. (2013). Preparation of organic Zn-phthalocyanine-based semiconducting materials and their optical and electrochemical characterization. Adv. OptoElectron..

[6] Rodríguez Gómez A., Sánchez-Hernández C.M., Fleitman-Levin I., Arenas-Alatorre J., Alonso-Huitrón J.C., Sánchez Vergara M.E. (2014). Optical Absorption and Visible Photoluminescence from Thin Films of Silicon Phthalocyanine Derivatives. Materials.

[7] Liu J.Y., Lo P.C., Jiang X.J., Fong W.P., Ng D.K.P. (2009). Synthesis and *in vitro* photodynamic activities of di-alpha-substituted zinc(ii) phthalocyanine derivatives. Dalton Trans..

[8] Kluson P., Drobek M., Kalaji A., Zarubova S., Krysa J., Rakusan J. (2008). Singlet oxygen photogeneration efficiencies of a series of phthalocyanines in well-defined spectral regions. J. Photochem. Photobiol. A.

[9] Iglesias R.S., Segala M., Nicolau M., Cabezo B. (2002). Computational study of the geometry and electronic structure of triazolephthalocyanines. J. Mater. Chem..

[10] Preethi N., Shinohara H., Nishide H. (2006). Reversible oxygen-binding and facilitated oxygen transport in membranes of polyvinylimidazole complexed with cobalt-phthalocyanine. React. Funct. Polym..

[11] Mafukidze D.M., Mashazi P., Nyokong T. (2016). Synthesis and singlet oxygen production by a phthalocyanine when embedded in asymmetric polymer membranes. Polymer.

[12] Pashkovskaya A.A., Perevoshchikova I.V., Maizlish V.E., Shaposhnikov G.P., Kotova E.A., Antonenko Y.N. (2009). Interaction of Tetrasubstituted Cation Aluminum Phthalocyanine with Artificial and Natural Membranes. Biochemistry.

[13] Reinhart T.J. (1987). Engineering Materials Handbook: Engineering Plastics Vol. 2.

[14] Caulfield B., McHugh P.E., Lohfeld S. (2007). Dependence of mechanical properties of polyamide components on build parameters in the SLS process. J. Mater. Process. Technol..

[15] KNF Corporation, Nylon Film Properties. http://www.knfcorporation.com/Nylon-products.html.

[16] Krishnan N.N., Henkensmeier D., Park Y.H., Jang J.H., Kwon T., Koo C.M., Kim H.J., Han J., Nam S.W. (2016). Blue membranes: Sulfonated copper(II) phthalocyanine tetrasulfonic acid based composite membranes for DMFC and low relative humidity PEMFC. J. Membr. Sci..

[17] Sánchez-Vergara M.E., López-Romero D.M., Vidal-García P., Jiménez-Jarquín C., Hernandez-García A., Jiménez-Sandoval O. (2017). Preparation of Hybrid Devices Containing Nylon/M(II)Pc-TTF (M=Cu, Zn) Films with Potential Optical and Electrical Applications. Electron. Mater. Lett..

[18] Schowalter S.J., Connolly C.B., Doyle J.M. (2010). Permeability of noble gases through Kapton, butyl, Nylon, and ‘‘Silver Shield”. Nucl. Instrum. Methods Phys. Res. A.

[19] Hammon H.G., Ernst K., Newton J.C. (1977). Noble gas permeability of polymer films and coatings. J. Appl. Polym. Sci..

[20] Habib B., Elham A., Ahmad B. (2016). Permeability of methane, carbon dioxide, oxygen and nitrogen gases using a composite membrane of polyethersulfone/polyamide 11. Int. J. Adv. Biotechnol. Res..

[21] Barzin J., Feng C., Khulbe K.C., Matsuura T., Madaeni S.S., Mirzadeh H. (2004). Characterization of polyethersulfone hemodialysis membrane by ultrafiltration and atomic force microscopy. J. Membr. Sci..

[22] Flaconnèche B., Martin J., Klopffer M.H. (2001). Permeability, Diffusion and Solubility of Gases in Polyethylene, Polyamide 11 and Poly(Vinylidene Fluoride). Oil Gas Sci. Technol..

[23] Le Huy H.M., Huang X., Rault J. (1993). Swelling and Deswelling of Polyamide 11 with Formic Acid. Polymer.

[24] Zugle R., Antunes E., Khene S., Nyokong T. (2012). Photooxidation of 4-chlorophenol sensitized by lutetium tetraphenoxy phthalocyanine anchored on electrospun polystyrene polymer fiber. Polyhedron.

[25] Zugle R., Nyokong T. (2013). Zinc(II) 2,9,16,23-tetrakis[4-(N-methylpyridyloxy)]-phthalocyanine anchored on an electrospun polysulfone polymer fiber: Application for photosensitized conversion of methyl orange. J. Mol. Catal. A.

[26] Zugle R., Litwinski C., Torto N., Nyokong T. (2011). Photophysical and photochemical behavior of electrospun fibers of a polyurethane polymer chemically linked to lutetium carboxyphenoxy phthalocyanine. New J. Chem..

[27] Modisha P., Nyokong T. (2014). Fabrication of phthalocyanine-magnetic nanoparticles hybrid nanofibers for degradation of Orange-G. J. Mol. Catal. A.

[28] Khoza P., Nyokong T. (2015). Visible light transformation of Rhodamine 6G using tetracarbazole zinc phthalocyanine when embedded in electrospun fibers and in the presence of ZnO and Ag particles. J. Coord. Chem..

[29] Goethals A., Mugadza T., Arslanoglu Y., Zugle R., Antunes E., Van Hulle S.W.H., Nyokong T., De Clerck K. (2014). Polyamide nanofiber membranes functionalized with zinc phthalocyanines. J. Appl. Polym. Sci..

[30] García-Moreno G. (2012). Organic Semiconductors Pi-Conjugated Based in Thiophene, Theoretical Study. Doctoral Thesis.

[31] López-Reyes M., López-Cortés J.G., Ortega-Alfaro M.C., Toscano R., Álvarez-Toledano C. (2013). First direct synthesis of 3-hydroxy-pent-4-ynoic acids. Application to the synthesis of pyran-2-ones. Tetrahedron.

[32] Sánchez-Vergara M.E., Leyva-Esqueda E.A., Álvarez C., López Reyes M., Miralrio A., Salcedo R. (2016). Influence of TCNQ acceptor on optical and electrical properties of tetrasubstituted allenes films fabricated by vacuum termal evaporation. J. Mater. Sci. Mater. Electron..

[33] El-Nahass M.M., Abd-El-Rahman K.F., Al-Ghamdi A.A., Asiri A.M. (2004). Optical properties of thermally evaporated tin-phthalocyanine dichloride thin films, SnPcC_l2_. Phys. B.

[34] El-Nahass M.M., Farag A.M., Abd-El-Rahman K.F., Darwish A.A.A. (2005). Dispersion studies and electronic transitions in nickel phthalocyanine thin films. Opt. Laser Technol..

[35] Karan S., Basak D., Mallik B. (2010). Persistence in photoconductivity and optical property of nanostructured copper (II) phthalocyanine thin films. Curr. Appl. Phys..

[36] Wang J.B., Li W.L., Chu B., Lee C.S., Su Z.S., Zhang G., Wu S.H., Yan F. (2011). High speed responsive near infrared photodetector focusing on 808 nm radiation using hexadeca-fluoro–copper–phthalocyanine as the acceptor. Org. Electron..

[37] Neghabi M., Zadsar M., Ghorashi S.M.B. (2014). Investigation of structural and optoelectronic properties of annealed nickel phthalocyanine thin films. Mater. Sci. Semicond. Process..

[38] Albo J., Hagiwara H. (2014). Structural Characterization of Thin-Films Polyamide Reverse Osmosis Membranes. Ind. Eng. Chem. Res..

[39] Albo J., Wang J., Tsuru T. (2014). Gas transport properties of interfacially polymerized polyamide composite membranes under different pre-treatments and temperatures. J. Membr. Sci..

[40] García J.M., Álvarez J.C., De la Campa J.C., De Abajo J. (1997). Synthesis and characterization of aliphatic-aromatic poly(ether amide)s. Macromol. Chem. Phys..

[41] Wagenpfahl A., Rauh D., Binder M., Deibel C., Dyakonov V. (2010). S-shaped current-voltage characteristics of organic solar devices. Phys. Rev. B.

[42] Jeon H.G., Ito Y., Sunohara Y., Ichikawa M. (2015). Dependence of photocurrent generation on the crystalline phase of titanyl phthalocyanine film in heterojunction photovoltaic cells. Jpn. J. Appl. Phys..

[43] Minami N. (1982). Photocurrent spectra of phthalocyanine thin-film electrodes in the visible to near-infrared. J. Chem. Soc. Faraday Trans. 2.

[44] Chawdhury N., Köhler A., Harrison M.G., Hwang D.H., Holmes A.B., Friend R.H. (1999). The effects of H_2_O and O_2_ on the photocurrent spectra of MEH-PPV. Synth. Met..

[45] Mark J.E. (1999). Polymer Data Handbook.

[46] Kayunkid N., Tammarugwattana N., Mano K., Rangkasikorn A., Nukeaw J. (2016). Growth and characterizations of tin-doped nickel-phthalocyanine thin film prepared by thermal co-evaporation as a novel nanomaterial. Surf. Coat. Technol..

[47] Pfeiffer M., Beyer A., Plönnigs B., Nollau A., Fritz T., Leo K., Schlettwein D., Hiller S., Wöhrle D. (2000). Controlled p-doping of pigment layers by cosublimation: Basic mechanisms and implications for theriuse in organic photovoltaic cells. Sol. Energy Mater. Sol. Cells.

[48] Iwase T., Haga Y. (2004). Photovoltaic characteristics of TCNQ-incorporated CuPc-poly(p-phenylene) composite films. J. Mater. Sci. Mater. Electron..

[49] Pfeiffer M., Fritz T., Blochwitz J., Nollau A., Plönnigs B., Beyer A., Leo K. (2007). Controlled Doping of Molecular Organic Layers: Physics and Device Prospects. Adv. Solid State Phys..

[50] Lapok L., Cyza M., Gut A., Kȩpcyński M., Szewczyk G., Sarna T., Nowakowska M. (2014). Synthesis, spectroscopic properties and interaction with a liposomal membrane of a novel iodinated magnesium phthalocyanine. J. Photochem. Photobiol. A.

[51] Azim-Araghi M.E., Krier A. (1997). Optical Characterization of Chloroaluminium Phthalocyanine (ClAlPc) Thin Films. J. Opt. A Pure Appl. Opt..

[52] Regimol C.C., Menon C.S. (2007). Effect of annealing and γ irradiation on tin phthalocyanine thin films. Mater. Sci.-Pol..

[53] Novotny M., Bulir J., Bensalah-Ledoux A., Guy S., Fitl P., Vrnata M., Lancok J., Moine B. (2014). Optical properties of zinc phthalocyanine thin films prepared by pulsed laser deposition. Appl. Phys. A Mater. Sci. Process..

[54] Wang H.M., Hsiao S.H., Liou G.S., Sun C.H. (2010). Synthesis, photoluminescence, and electrochromism of polyamides containing (3,6-di-tert-butylcarbazol-9-yl)triphenylamine units. J. Polym. Sci. A.

[55] Liou G.S., Huang N.K., Yang Y.L. (2006). Blue-light-emitting and anodically electrochromic materials of new wholly aromatic polyamides derived from the high-efficiency chromophore 4, 40-dicarboxy-400-methyltriphenylamine. J. Polym. Sci. A.

[56] Li X., Zhu H., Wei J., Wang K., Xu E., Li Z., Wu D. (2009). Determination of band gaps of self-assembled carbon nanotube films using Tauc/Davis–Mott model. Appl. Phys. A Mater. Sci. Process..

